# Revisiting Deception in Breonna Taylor’s Case: A Cognitive-Acoustic Approach

**DOI:** 10.1007/s10936-023-09956-1

**Published:** 2023-04-07

**Authors:** Amr M. El-Zawawy

**Affiliations:** Faculty of Education, Alexandria University, Alexandria, Egypt

**Keywords:** Cognitive load, Deception, Breonna Taylor, Lie, Detection

## Abstract

The present paper proposes an eclectic model for examining the cognitive load involved in detecting deception that benefits from the acoustic dimension as an exercise in cognitive forensic linguistics. The corpus used is composed of the legal confession transcripts of the Breonna Taylor’s Case, a 26-year-old African-American woman worker who was shot dead by police officers in Louisville, Ky., in March 2020 during a crackdown on her apartment. The dataset comprises transcripts and recordings of the persons involved in the shooting event but have given unclear charges, and those accused of contributory negligence due to wanton misfiring. The data is analyzed based on the video interviews and reaction times (RT) as an application of the model proposed. The findings reveal that the episodes chosen and how they are analyzed exhibit that the modified ADCM along with the acoustic dimension provide a clear picture of cognitive load management in the course of constructing and producing lies.

## Introduction

As defined by Zuckerman, DePaulo, and Rosenthal (1981, p.4), deception is ‘an act that is intended to foster in another person a belief that which the deceiver considers to be false.’ This broad definition is rather significant, since it entails the themes of belief, falsehood and the process of ‘fostering’ this belief in the receptor by means known to the sender. This definition also points to the notion of lying as integral to deception, and this is the focus of the present paper.

The question of how lies are constructed and delivered is both confusing and confused. The double confusion stems from the fact that the process of constructing lies is both linguistically and cognitively grounded (see below the section on verbal and non-verbal cues), which means that cognition operates to provide a dubious message and at the same time linguistically well-formed. The process is cognitively taxing and includes a certain load to be handled by the sender carefully. This ‘handling’, so to speak, is worthy of research, given the conflicting body of literature on lie detection, where naturalistic versus laboratory-induced investigations call for discrete methods and approaches.

The term ‘cognitive load’ can be defined as a multidimensional construct representing the load imposed by performing a particular task on someone’s cognitive system (Paas et al., 2003; Paas & Van Merriënboer, 1994; Paas, F., Tuovinen, J. E., Tabbers, H., & Van Gerven, P. W., 2016). A number of models have been proposed to account for the cognitive load involved in producing lies, and some of them have been empirically justified (see Walczyk et al., 2003), yet there is a clear tendency to isolate factors that otherwise would greatly assist the investigation of cognitive load during lying and provide a more detailed picture of that effort. Among these factors is the acoustic dimension of lying (including but not limited to RT, F0, and intonation) which provides valuable data that sometimes (in)validates the findings.

The present paper proposes an eclectic model of examining the cognitive load involved in detecting deception that benefits from the acoustic dimension and even justifies the data analyzed. The corpus used is composed of transcripts of the confessions of the Breonna Taylor’s Case, a 26-year-old African-American woman worker who was shot dead by police officers in Louisville, Ky., in March 2020 during a crackdown on her apartment. The dataset comprises transcripts and recordings of the persons involved in the shooting event with unclear charges and those accused of contributory negligence due to wanton misfiring. The data is analyzed based on the video interviews and reaction times (RT) as an exercise in cognitive forensic linguistics.

## Lie/Deception Detection

### A Brief Review

The problem with the literature on lie detection is the myriad of methods and models adopted in the course of deciding whether the sender is lying (Porterand and Brinke, 2010). In general terms, there are two approaches that can be outlined: one considers lying as a natural phenomenon that can be explained by means of rigid models and examined under laboratory conditions. This subsumes exposing participants in an experimental design to a number of situations or actions to calculate and tabulate statistical results justifying certain cues. This approach even requires participants to recall events in reverse order, which is very rare in everyday locutions where lies occur (Vrij & Granhag, 2012). The other approach uses naturalistic data to see whether lies are there or not. This may also be geared towards discovering cue prominence or engage in lengthy statistical analyses that pinpoint the specifics of lying, whether verbal or non-verbal. A selection of the most prominent studies in each approach will be presented below, followed by the cognitive load studies which are not given due attention in the literature on deceptive speech.

An early attempt among the first approach is Zuckerman et al’s (1981). They attached much attention to the meta-analysis of deception-detection (traditionally known as the Four-Factor Theory), and stated that no cue or cues to deception could be accurate all the time because deception was an individual psychological process. The four factors are as follows: emotional reactions, cognitive effort, attempted behavioral control and arousal. Zuckerman et al. (1981) maintained that a deceiver must be engaged in all these four factors at once while composing deceptive discourse.

Another chief model is the IDT (Interpersonal Deception Theory) (Buller & Burgoon, 1996; Zhou et al., 2004a), which describes an iterative process of mutual influence in which the deception by one conversational participant provokes a series of moves and countermoves by both parties to the conversation. These moves are aimed on the one hand at adapting the deceptive message in order to maintain its apparent truthfulness (i.e., achieving deception success) and on the other at discerning the credibility of the message and the sender and ultimately reaching an interpretation of the meaning (i.e., achieving detection success). As El-Zawawy (2017) notes, Burgoon et al. (2012) focused on whether indicators of truth or deception are context-independent or context-sensitive. The factors they suggested are: motivation and modality. A 2 (veracity: truthful/deceptive) by 2 (incentives: high/low) by 3 (modality: FtF/audio/text). Their factorial experiment revealed that linguistic indicators are significantly related to veracity, but the results are highly sensitive to context.

Hartwig and Bond (2014) adopted meta-analysis to assess the detectability of lies from constellations of multiple cues, with a particular focus on whether lie detection increases as the conditions approach real-life. They synthesized 144 samples, including 9380 liars and truth tellers providing a total of 26,866 messages. Their findings show that lies can be detected with nearly 70% accuracy. This level of detection was stable across settings.

Reynolds and Randle-Short’s (2011) adopted a rigorous methodological framework of conversation analysis (CA) as analytic tool kit to demonstrate the importance of context, paying extra attention to cues to deception in order to understand whether there is a relationship between response latency and deception. Reynolds et al. examined data from outside laboratory settings taken from *The Jeremy Kyle Show*. They selected certain criteria based on how participants in the outside-laboratory interactions formulate their verbal output. Lies were detected according to the following criteria: (1) agreement by the liar that a lie had occurred; (2) explicit labeling of talk as lies by other participants; and (3) the liar’s ‘revision’ of a prior action, thereby changing the.

The second broad approach is characterized by inducing certain cues under laboratory conditions. One significant study in this regard is Upchurch and O’Connell’s (2000).They examined the recordings of ten excerpts of both President Clinton’s Grand Jury Testimony of August 17, 1998 and of each of two interviews with Hillary Rodham Clinton (*Today Show,* NBC, January 27, 1998; *Good Morning America,* ABC, January 28, 1998). Longer excerpts were selected for purposes of reliability and full response to the interviewer. The statement of innocence, made at the conclusion of an educational press conference on January 26, 1998, and the formal admission of involvement with Ms. Lewinsky, made at the beginning of the Grand Jury Testimony, were also analyzed. Comparisons were then  made with other modern presidential inaugural rhetoric. Their study revealed that dramatic performance of the interviewees greatly affected the veracity of their statements.

Another significant study is Demenko’s (2008). She introduced voice stress extraction and classification into the investigation of deceptive speech. Her study utilized of the authentic Poznan police database which  contained recordings of the 997 emergency phone calls, and selected 20,000 recordings out of 60,000, out of which around 100 were acoustically analyzed. It was concluded that the range of fundamental frequency per se did not correlate with stress whereas the shift in fundamental frequency register constituted the primary indicator of stress. Through Linear Discriminant Analysis based on 12 acoustic features, the study revealed it is possible to reach the three categories of neutral, depressive, stressed, highly stressed speech.

In a similar vein, Kirchhübel and Howard (2011) discussed acoustic changes in deceptive statements. Their dataset included truthful, deceptive and control speech from ten speakers during an interview. Results were displayed according to the parameters of F0, intensity and vowel formants. The study revealed that no significant correlation could be established for any of the acoustic features, a result that runs counter to many mainstream studies in the field.

El-Zawawy (2017) examined how the two US presidential candidates Donald Trump and Hillary Clinton use statements judged to be false by the Politifact site while delivering their campaign speeches. The corpora used contained some statements accompanied by the video recordings. Based on CBCA (Criteria-based Content Analysis), a new linguistic model was proposed, and the data were analyzed using software, namely LIWC (Linguistic Inquiry & Word Count), and also focused on the content analysis of the deception cues that can be matched with the results obtained from computerized findings. When VSA (Voice Stress Analysis) was required, PRAAT 6 was used. The study concluded that the New Model (NM) is not context-sensitive, being a quantitative one, and is thus numerically oriented in its decisions. Moreover, when qualitative analysis intervenes, especially in examining Politifact rulings, context plays a crucial role in passing judgments on deceptive vs. non-deceptive discourse.

Still, there are other studies that addressed the cognitive dimension of lie detection, but the literature on them is parsimonious. Although they mostly follow the trend of inducing responses under laboratory conditions, they are not as prominent as cue extraction and validation studies. They can actually be subsumed under Zuckerman et al’s (1981) ‘cognitive effort’ (see above). One of the key models in this respect is Walczyk et al’s (2003), where ADCM (Activation-Decision-Construction Model) is proposed as a detailed operational model to map the cognitive process of lying. The ADCM is built on constructs from Baddeley’s Working Memory model (1992). The Activation component is concerned with the recall of the truth in long-term memory, which is then transferred to and stored in the working memory. During the Decision process, deciding to produce a lie is based on whether answering truthfully is in the sender’s self-interest. The decision to lie guides the central executive (an attention-controlling system) to conceal the truth. The Construction component is based on the Construction- Integration Model (Kintsch, 2004). Although orderly and attractive, the model was criticized for its lack of detail and for the linearity according to which it operates. The model was also validated in the laboratory within a sample of around 200 participants, but with no or scant naturalistic data.

From the brief review above, it is clear that the body of literature on lie detection still lacks a unified approach that relies on naturalistic data without human intervention. This points to the gap of combining several models and approaches, supported by acoustic evidence, to provide a more plausible picture of how cognition, particularly cognitive load, operates and can be utilized to be a viable extra tool for lie detection. This is the task in the present paper.

### Verbal (Linguistic) Cues

The above approaches provide the umbrella under which other discrete studies attempted to zoom in on certain specifics of deceptive speech, particularly cues, to avoid conflicting findings, though most deception cues proved to be faint and unreliable (Adams-Quackenbush, 2015). Traditionally, these studies classified cues to deception/lying into verbal vs. non-verbal ones. This section is concerned with verbal cues. According to Picornell (2013), verbal/linguistic cues are the following:

1. Word Quantity
2. Pronoun Use
3. Emotion Words

However, other researchers, especially Jelveh (2015) and Fitzpatrick et al. (2015), provide the following:

1. Word quantity: the calculated number of words.
2. Inconsistency and contradiction: as related to the content of the analyzed data.
3. Generalization: broad descriptions of the events.
4. Contraction of negative sentences: shortened negative markers or content.
5. Emphatic use of language: using more polite expressions.
6. Vagueness of statements: unclear explanations.
7. Use of negative emotion words: such as ‘don’t like’, ‘don’t feel’, etc.
8. Deflection: a defense in which someone blames you for something they are at fault for.
9. Past to present tense shift: avoiding consistency in time reference.
10. Use of specific words which can reveal opinions (e.g. whatever: contempt or if: self doubt).
11. Excessive use of hedges and modifiers: such as ‘as far as’, ‘to some extent’, etc.
12. Group references vs. self-references: ‘we’ vs. ‘I’.
13. Repetition to buy time: lengthy accounts with repeated content.
14. Excessive use of gap fillers (e.g. actually, etc.).

These cues are usually validated by statistical means based on certain experimental designs. Yet only a relatively small scientific community of linguists, psychologists, and computer scientists deal with verbal cues to deception (cf. Fitzpatric et al., 2015).

### Non-Verbal Cues

Non-verbal cues include the ones that give details about the voice and body movements. According to Fitzpatrick et al. (2015), they subsume the following:

1. Voice f0
2. Filled/silent pauses
3. Disfluencies
4. Microexpressions
5. Pupil dilation
6. Finger tapping

## Police Interrogations as Naturalistic Data

As stated in the Introduction, the present paper investigates the case of Breonna Taylor, where the police interrogations furnish the body of the corpus analyzed. It is important to underline how police interrogations can provide valuable datasets that are primarily naturalistic.

As Gaines and Lowrey-Kinberg (2020, p.128) state, ‘less work has been done by linguists and discourse analysts on the language of interrogation’. Yet one of the most well-known models of police interviews is the PEACE model. As Rock (2020) explains, the PEACE model is based on techniques from cognitive interviewing and conversational management. The model proposes that investigative interviewing, as opposed to interrogation, depends on a specific set of activities and skills. The concepts underlying the model can be divided into the following steps:

1. **Planning and preparation**: Takes place before the interview begins and involves activities like making notes about legal topics such as points to prove and identifying any logistical needs.
2. **Engage and explain**: Describes the opening phases of an interview during which the officer will explain the upcoming interview procedure.
3. **Account**: Denotes the main ‘questioning’ sequence and therefore has obvious relevance to texts produced during planning and preparation. The officer will both use notes written before the interview and make notes for further questioning or subsequent investigation.
4. **Closure**: Provides both formal termination of the interview, as the officer explains legally required matters.
5. **Evaluation**: Post- interview assessment at this stage provides both a platform for the officer’s personal and professional development and, in relation to the investigation itself.

However, no study to date has applied this model outside the courtroom in linguistic and non-linguistic contexts and with no view to detecting deception. As Haworth (2020) maintains, police interview data undergo various changes in format, raising serious questions about their reliability. She questions the validity of police interviews as faithful representations of the interviewee’s actual accounts.

Yet the advantage embedded in such interviews is their nature: they are raw data that can be of value to investigations into lying. The problem of their dubiety can be addressed by means of other combinatory methods to compensate for any shortcomings. These methods include the utilization of acoustics and RT as a valid tool for tracing cognitive load, which is one of the main objectives of the current study.

## Context of the Problem

In March 2020 during a crackdown on her apartment, Breonna Taylor, a Black medical aide who was shot dead by police officers in Louisville, Ky. The incident triggered wide-scale demonstrations that year over policing and racial injustice in the United States.

On Aug. 4 of the same year, the Justice Department charged four current and former police officers with federal civil rights violations, including lying to obtain a search warrant for her apartment. Out of a number of detectives, including Kelly Goodlett, who retired after being charged and pleaded guilty, and Kyle Meany who was fired by the Louisville Police Department on Aug. 19.

Brett Hankison, a third officer facing the federal charges, was the only officer to face state charges in the raid. He was accused of wanton endangerment of neighbors whose apartment was hit when he fired into Ms. Taylor’s apartment. He pleaded not guilty and was acquitted.

On Aug. 4, prosecutors said that three officers, Joshua Jaynes, Kelly Goodlett and Kyle Meany, provided false affidavits to obtain the search warrants and conspired to lie about it after. The affidavits claimed that Kenneth Walker, Taylor’s ex-boyfriend, had been receiving packages at her address, which was not true.

Taylor’s family disputed the police’s claim that the raid had to be conducted in the middle of the night. Their lawyers stated the officers proceeded to shoot indiscriminately into her residence.

Hankison was fired from the Louisville Metro Police Department in June after investigators found he had wantonly and blindly fired 10 times during the raid, according to his termination letter. Both Mattingly and Cosgrove were reassigned to administrative duties.

As the event is complex and includes several detectives, the present paper focuses on the three officers/detectives, namely Brett Hankison, Jonathan Mattingly and Myles Cosgrove, since they were relieved from their jobs in connection with the shooting incident, which was the main cause for the row that ensued.

The paper applies an eclectic model of investigating the cognitive load involved in detecting lies and deception can benefit from the acoustic dimension and even justify the data analyzed.

## Methodology

### Corpus

A corpus of the recordings of three detectives involved in the death of Breonna Taylor was compiled. It consisted of 10 episodes, where the detectives were being interviewed. The episodes were selected based on their significance to the shooting incident of Breonna Taylor, either by her boy friend Kenneth Walker or by the three detectives. The Tables 1, 2, and 3 summarize the details of the episodes and their types.

**Table 1:** A summary of the details of the episodes and their types

Number of episodes analyzed	Detective	Duration in minutes (excluding interrogators’ questions)	Type: monologue or dialogue
3	Brett Hankison	51:43	3 monologues
4	Jonathan Mattingly	39:03	3 monologues, 1 dialogue
2	Myles Cosgrove	58:20	1 monologue, 1 dialogue
2	Kenneth Walker	58:44	2 monologues
Total	4	4.43 h	9 monologues 2 dialogues

**Table 2:** The means of RT for each of the detectives in addition to Kenneth Walker

Detective’s name	RT mean in milliseconds
Brett Hankison	619
Jonathan Mattingly	437
Myles Cosgrove	394
Kenneth walker	308

**Table 3:** The RT means compared with the durations of each detective’s accounts

Detective’s name	RT mean in milliseconds	Length of Account in minutes	Length of Account in milliseconds
Brett Hankison	619	51:43	51,430
Jonathan Mattingly	437	39:03	39,030
Myles Cosgrove	394	58:20	58,200
Kenneth Walker	308	58:44	58,440

The interrogators’ questions have been deducted from the calculated time

The corpus is thus all made up of naturalistic data without any laboratory-induced effects.

#### Preparing the data for analysis

The recordings downloaded from the Internet were transcribed verbatim and checked against the PIU (2019) obtained from the official website of Louisville Police, and any omissions and/or additions were noted and/or incorporated. The material was filtered to reduce noise, especially background noise and hums and hisses at a − 28 dB rate with a multi-band noise utility by WavePad Sound Editor 10.4.

### Methods of Analysis

#### Modified Model of Analysis

In producing deceptive discourse, liars have to fulfill three demanding tasks. First, they have to provide narratives different from the truths they entertain. Second, they also have to monitor the plausibility of their statements and avoid contradictions in their stories. Finally, they have to appear undisturbed and poised in order to avoid agitation and collapse. These steps are very cognitively taxing, since they demand extra efforts that affect their RT^1^ and acoustics.

**Fig. 1:**
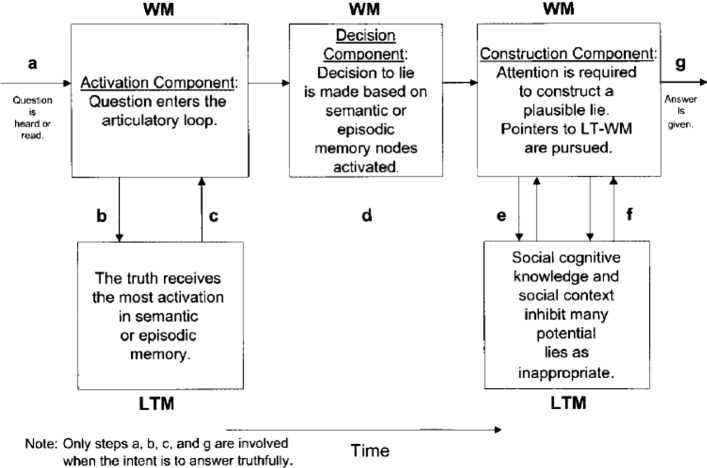
The activation-decision-construction model (Walczyk et al.’s., 2003)

One major approaches to investigating the field of lie-detection is the ADCM (Activation-Decision-Construction Model)^2^ which has proposed as a detailed operational model to map the cognitive process of lying. Figure 1 provides a graphic representation of the model.

The ADCM is built on constructs from Baddeley’s Working Memory model (1992). The Activation step is concerned with the recall of the truth in long-term memory, which is then transferred to and stored in the working memory. During the Decision step, the decision to lie is based on whether answering truthfully is in the sender’s self-interest. This decision causes the central executive (an attention-controlling system) to conceal the truth. The Construction step is based on the Construction- Integration Model (Kintsch, 2004).^3^ Although orderly and attractive, the model was criticized for its lack of detail and for the linearity according to which it operates. The model was also validated in the laboratory within a sample of around 200 participants, but with no or scant naturalistic data.

The model in its 2003 version was preferred to the later version Activation-Decision-Construction-Action (ADCA), which is mainly based on feelings and impressions stirred by the speaker’s output as a way to utter or withhold a lie. This last point is not the focus of the present paper. Thus, the 2003 model is more amenable to application to the present dataset, but to eschew its linearity, a modification is introduced. This entails adding backtracking and looping as two components that make the model more flexible. Backtracking includes the possibility to reverse to an earlier stage or step and jump forward without passing through all the steps. Looping entails a buffering movement from one component to another, namely from LTM to WM components, in either direction to facilitate cognitive flexibility. The present model of ADCM actually has this feature but only in one direction and in an imbricated manner, where there is no direct move from step a to steps e and f, or from steps b and c to g. Looping is confined to the articulatory input, and does not include from b and c to g or from a to e and f.

**Fig. 2:**
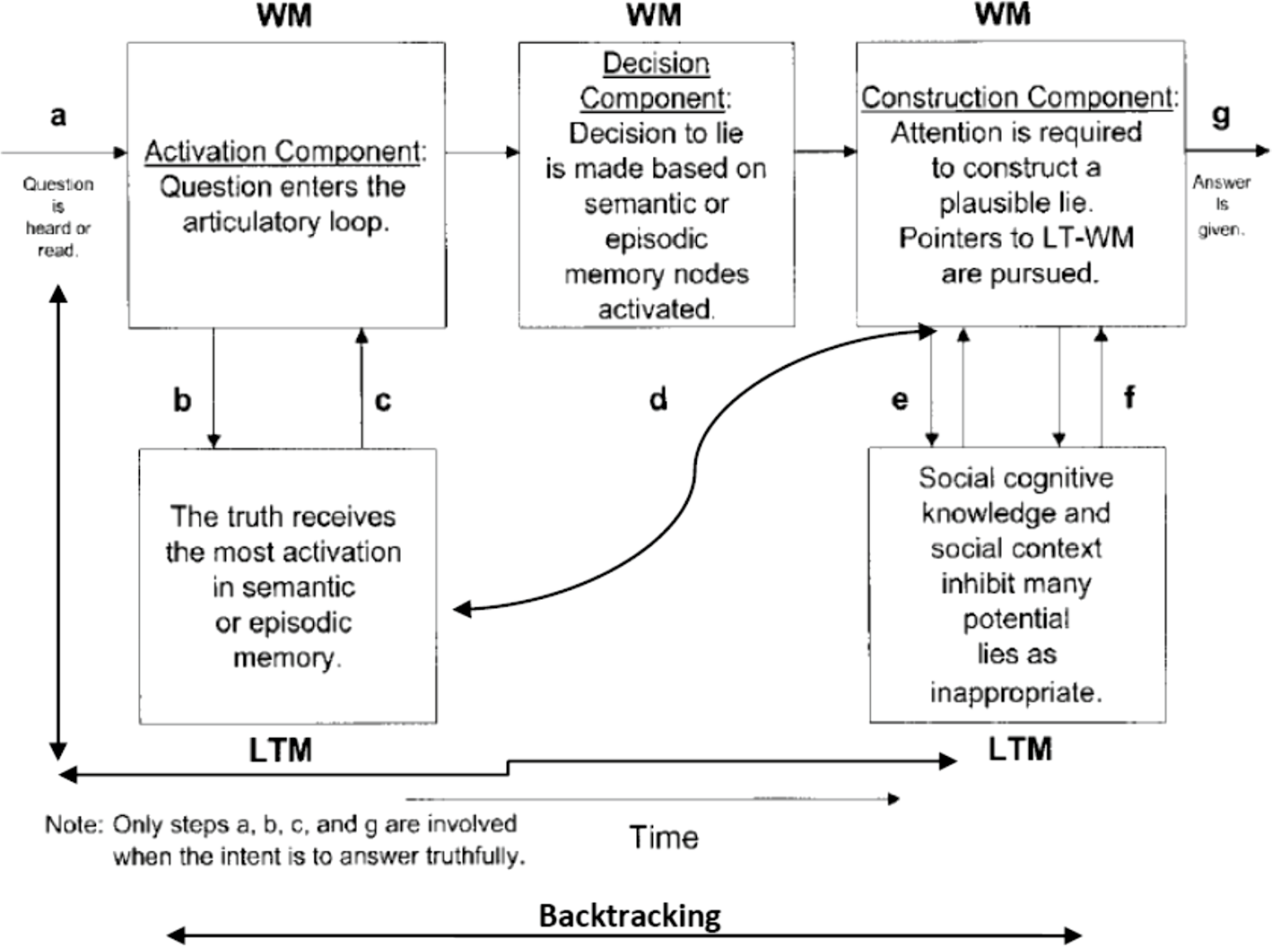
A modified activation-decision-construction model

These two features contribute to explaining more how lying and prevarication happen. Combined with RT rates and F0 and occasional pause detection, it can also provide a more plausible picture about how cognitive load is managed in the course of producing truths and lies and can act as viable VSA (Voice Stress Analysis) tools. The new model can be visually represented in Fig. 2.

The analysis focuses on the ADCM of the problematic parts in the episodes selected, and a visual representation is provided when necessary. Spectrograms illustrating F0 and pitch variations are produced by PRAAT 6 to match the ADCM with the acoustic dimension. Sometimes cross comparisons are made to corroborate certain assumptions. The analysis is divided into the examination of three categories:

1. Officially charged detectives.
2. Inconclusively charged detective or those with unclear charges.
3. Overall analysis of reaction times (RT)

The third category is added to project a complete image of the accusations and to integrate the statistical analysis dimension, which is of benefit to comparing certain datasets in the present corpus.

## Analysis of the Data

### Officially Charged Detectives’ Episodes

This includes the interrogation of Brett Hankison, who was given a termination letter, based on his wanton use of fire. The episodes of his answers where he spoke about shooting were examined, since they testify to his use of gunfire without restraint. Each episode is provided with its timestamps according to the recording of the interrogation by Sgt. Amanda Seelye.

Episode 1:

13:57 and that's when I saw darkness in the

14:00 apartment but then I saw an immediate

14:02 illumination of fire

14:06 come and what I saw at the time was a

14:09 figure

14:10 in a shooting stance and it looked as if

14:13 he was holding

14:15 he or she was holding an ar-15 or a long

14:18 gun a rifle

Here, Hankinson describes his encounter with the person who was shooting at the police, probably Breonna’s boyfriend, Kenneth Walker, who testified he released one shot as a warning from his licensed gun. This came as a lengthy answer to a question by the interrogator asking for a description of the shooting encounter. According to the ADCM, Hankison engages first in the Activation phase, and recalls from the LTM the semantic components of the encounter in addition to the social and cognitive context in order to appear as truthful as possible. Yet this data can be matched with what Kenneth Walker mentioned in his testimony, where he stated the following:

So I just let off one shot like I can’t still see who it is or anything. So now the door’s like flying open. I let off one shot and then all of a sudden there’s a whole lot of shots.

**Fig. 3:**
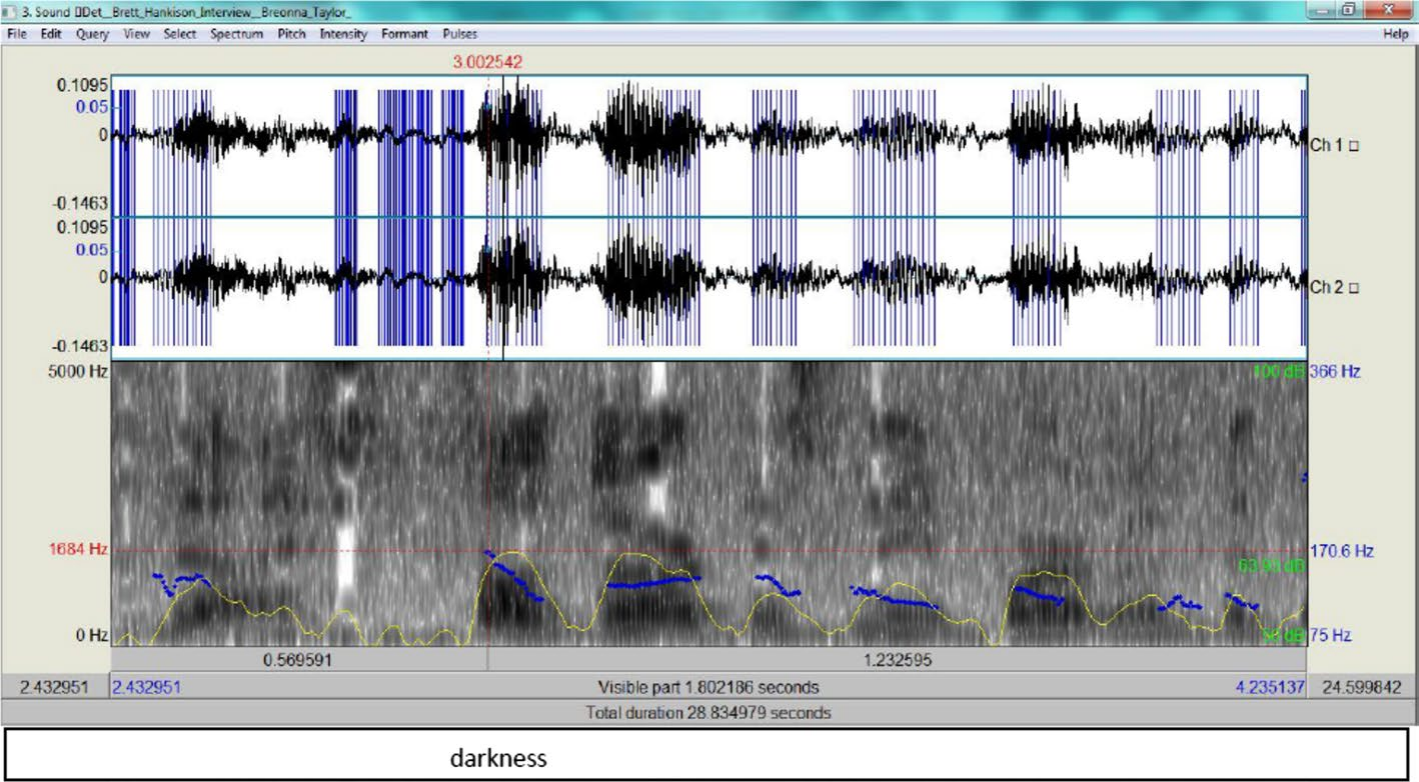
The spectrogram for ‘I saw darkness’

Walker’s statement can be taken as the social and cognitive context which Hankison composed his testimony against. What can be used here to determine whether Hankison is being deceitful is the RT plus his intonation. According to PRAAT 6 software, the episode was preceded by some pause, then Hankison continued. The RT is estimated to be 1,400 ms, which is significant because, according to Walczyk et al. (2003) (see also Verschuere et al., 2018), where a response would be produced in less than 400 ms, and lies consume approximately 200 ms longer to produce (600 ms in total). This also indicates the heavy cognitive load he was experiencing. Here the difference is clear and the PRAAT waveform spectrogram provides Fig. 3 for ‘I saw darkness’.

**Fig. 4:**
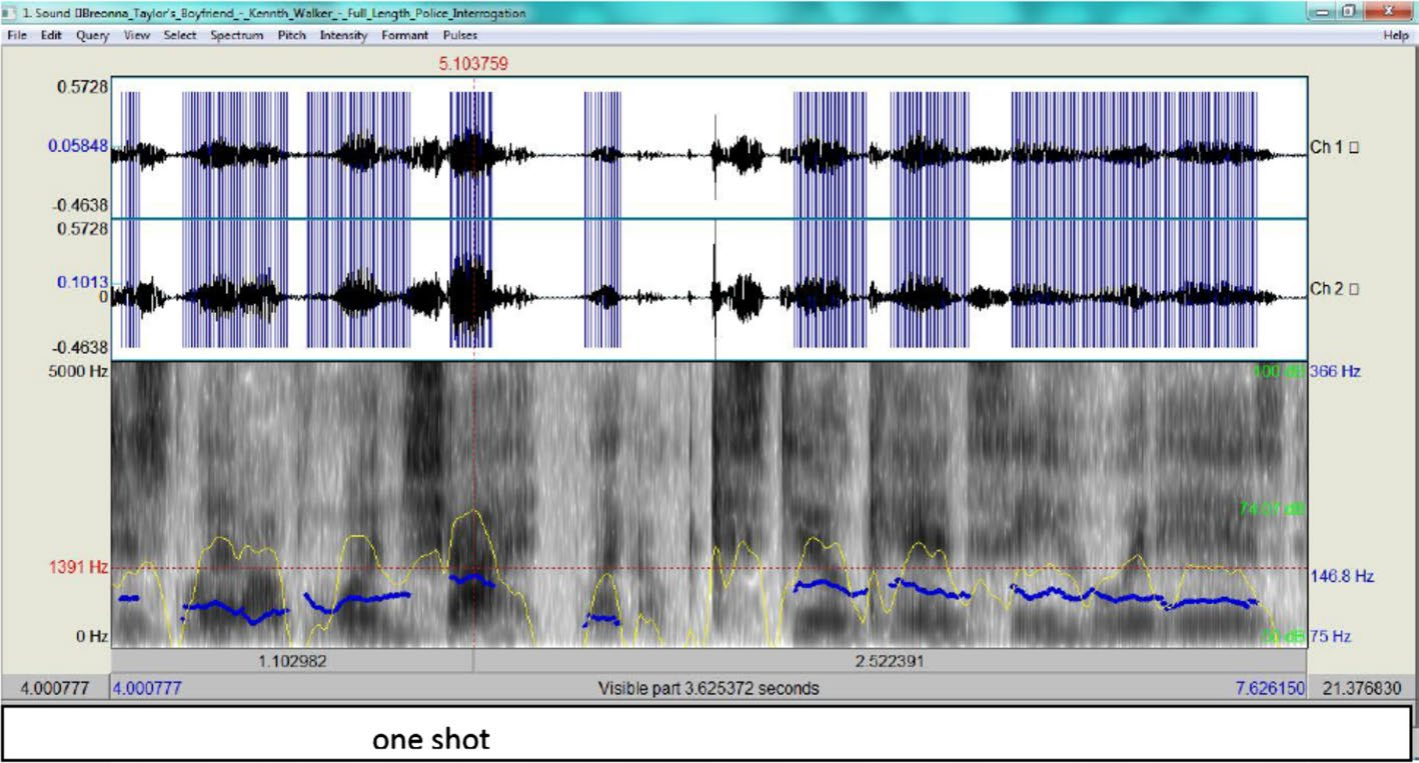
The spectrogram for ‘one shot’

**Fig. 5:**
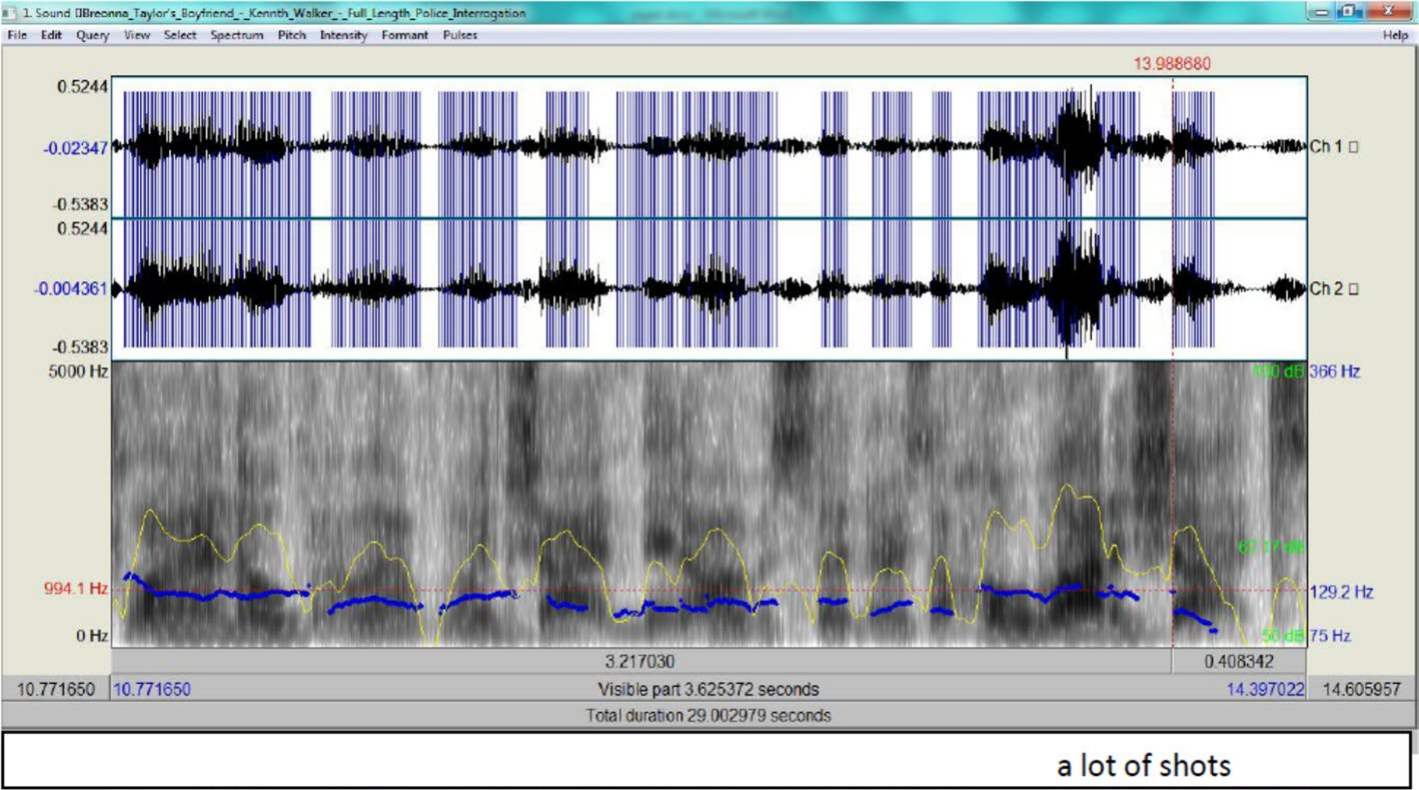
The spectrogram for spectrogram for ‘a lot of shots’

From Fig. 3, it is clear that the intonation marks a stress in his delivery (the pitch contour as an indicator of F0 is 170.6 Hz, that is almost half way up to the upperbound 366 Hz)and the blue streaks exhibit how high tone is rise-fall which betrays some tension on his part. Figure 4 provides the spectrogram for 'one shot' then is compared to Walker's in Fig. 5.

According to Fig. 4, Walker’s tone is clearly more stable (the pitch contour as an indicator of F0 is 146.8 Hz) as shown by the blue streaks marking. This is within the male normal range, i.e. low 65 Hz and high 366 Hz (see Scherer et al., 1985 and Demenko, 2008). His RT is also normal . Walker’s spectrogram for ‘a lot of shots’ is in Fig. 5.

From Fig. 5, Walker’s tone is also stable and falling, which conveys an attitude of certainty (the pitch contour as an indicator of F0 is 129.2 Hz)as shown by the blue streaks marking. This is within the male normal range, i.e. low 65 Hz and high 366 Hz (see Demenko, 2008).

A further analysis of Hankison’s description of what he saw reveals that first he said ‘figure’ then ‘he or she’: he inserted the pronoun ‘she’ to indirectly refer to ‘Breonna Taylor’, though she never owned a gun. The spectrograms of these two phrases show how his tension is building up.

**Fig. 6:**
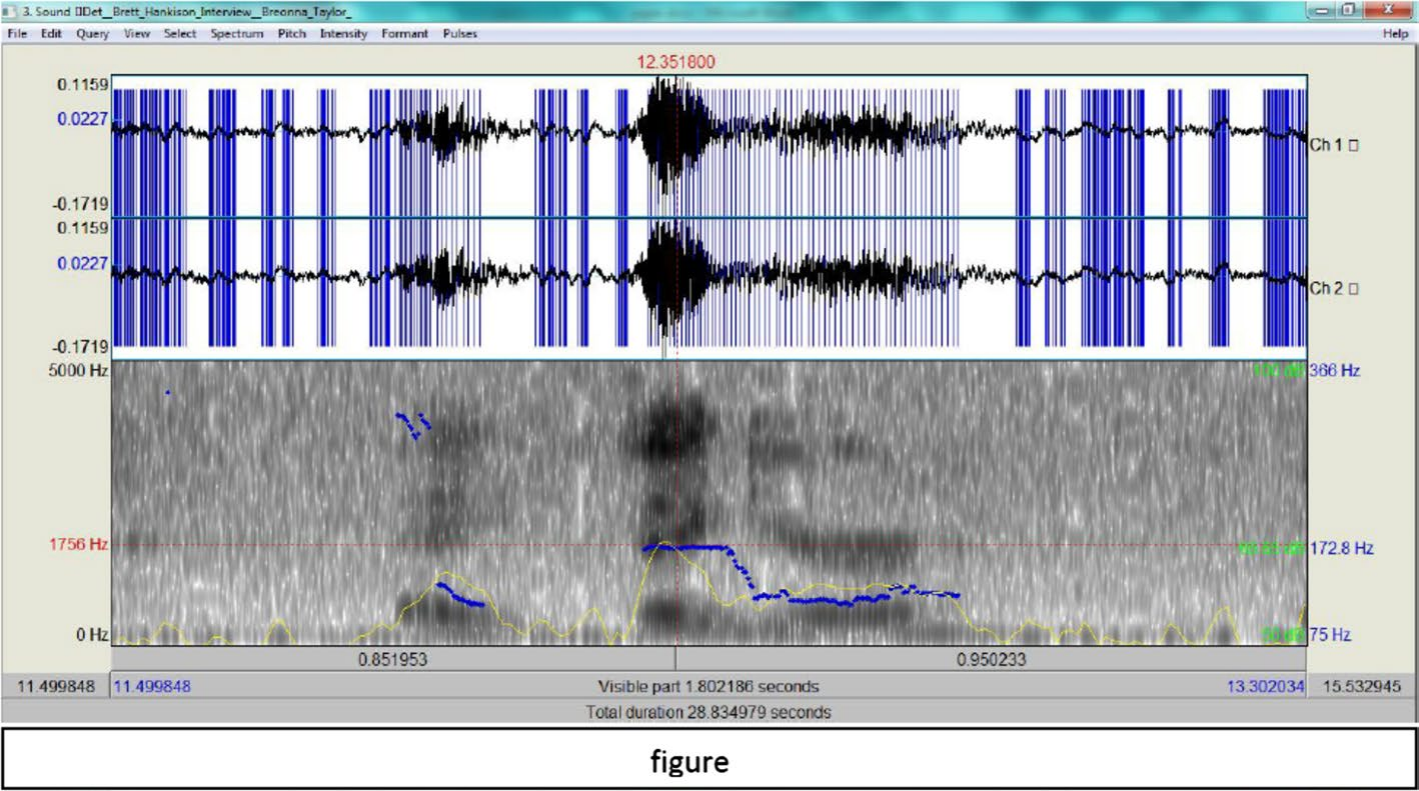
The spectrogram for ‘figure’

In Fig. 6, Hankison's tone is falling then suddenly it rises and becomes level then falls. Even when it is level, it marks an F0 of 172.8 Hz, which indicates an insistence on being unclear. Hankison’s spectrogram of ‘he or she’ is also indicative.

**Fig. 7:**
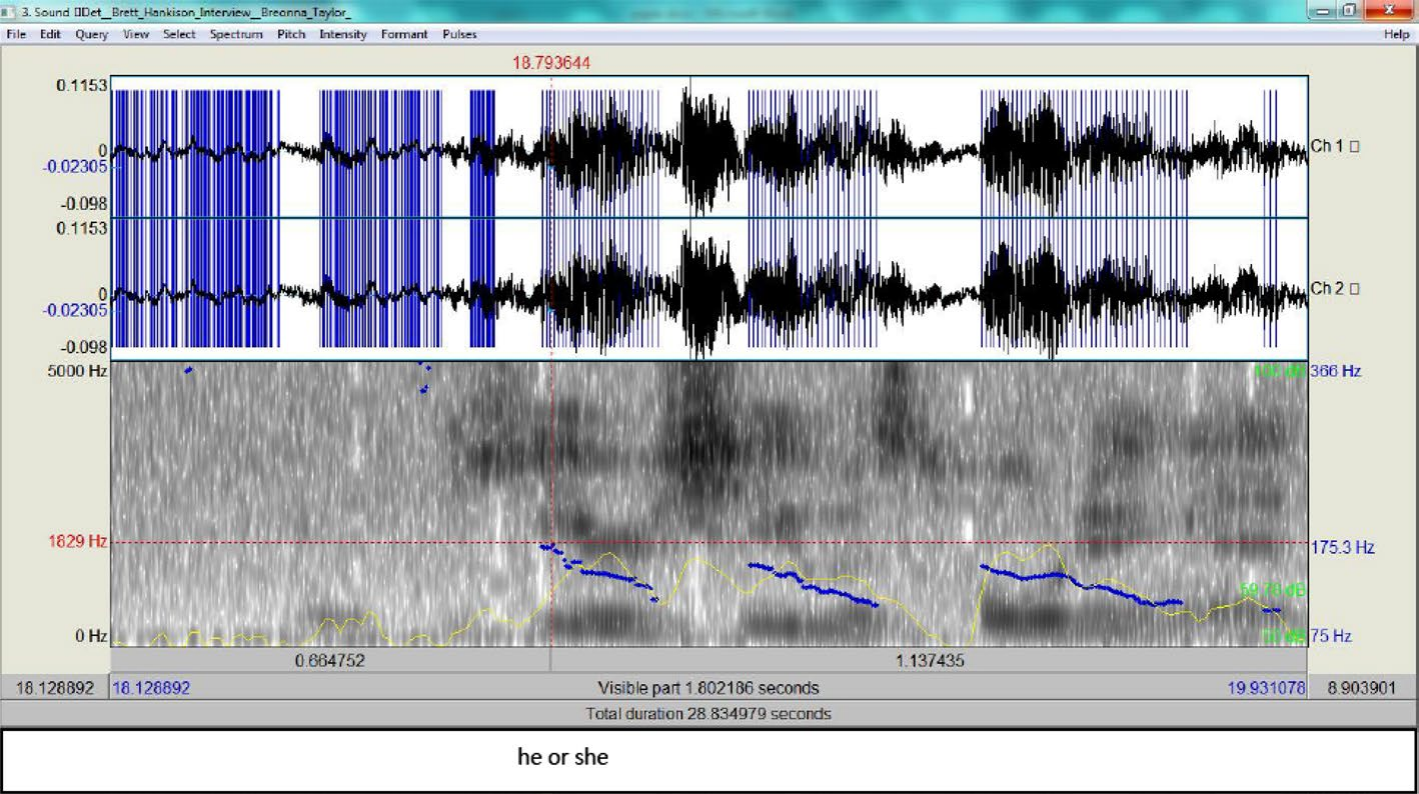
The spectrogram for ‘he or she’

**Fig. 8:**
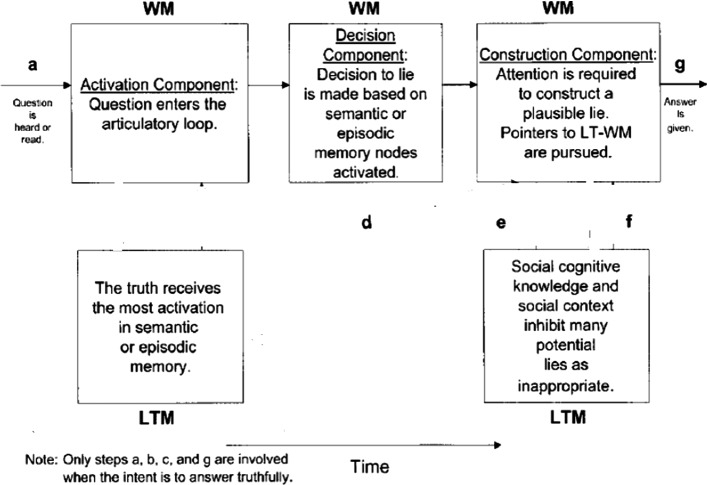
The ADCM for Hankison’s statement in the first episode

According to Fig. 7, the peak of the pitch level is at ‘she’, which may indicate that Hankison has inserted this pronoun through step e in the ADCM. This means that, according to the ADCM, he has managed to provide a fabricated account, since the steps a, d and g without attempting steps b, e, and f where truth is checked (Fig. 8).

Episode 2:

14:39 like like we would if we were at the

14:41 range or the best way to describe it is

14:44 to me when I guess on my mind process it

14:46 was it was we were at the range shooting

14:48 targets and you know how they will have

14:49 to literally have the targets

14:51 turn and it's either a bad guy or a good

14:52 guy you don't know and they'll threaten

14:55 and it was literally I saw that threat

14:59 target and then the muzzle flash from

15:02 the from the gun

**Fig. 9:**
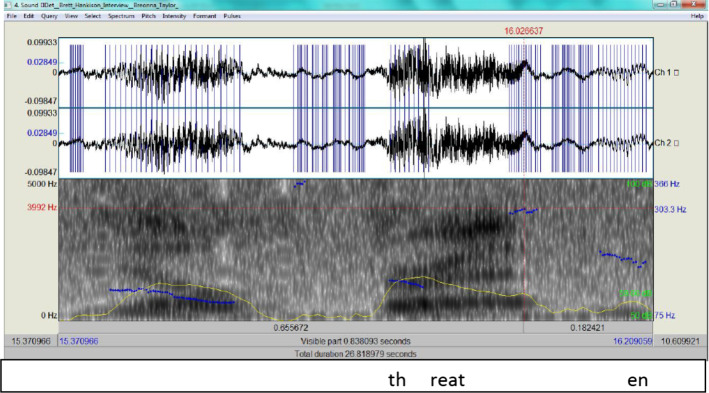
The spectrogram for ‘threaten

Here, Hankison described in more detail how the shooting encounter happened, and he justified his random firing. His account relies on engaging the interrogator with him by using the pronoun ‘you’. Thus, his cognition, according to ADCM, operates according to steps d and e before going to g. This also runs counter to his statement above when he said, “(14:13) he was holding (14:15) he or she was holding an ar-15 or a long (14:18) gun a rifle”. How come he could not figure out clearly who was shooting, as he just saw a flash from the muzzle, when he could give a specific size for the rifle? This is corroborated by the spectrogram for ‘threaten’ in Fig. 9.

It is clear from Fig. 9 that the intonation marks a stress in his delivery and the blue streaks exhibit how high tone is rise which betrays extreme agitation on his part (the pitch contour as an indicator of F0 is 303.3 Hz) (see Scherer et al., 1985). There is no clear justification for being tense when detailing a past experience. It can be an attempt on his part to influence the receiver/interrogator and appear emotional to justify his random firing later on. Hankison’s account is clearly a result of excessive cognitive overload, and the veracity of his description is highly questionable.

Episode 3

17:14 a rifle I thought I saw someone in a

17:16 shooting stance with the rifle you know

17:17 with the left hand on the

17:19 on the gun and bracing it on their

17:21 shoulder

17:22 and squatted down like in a military

17:24 tile style shooting stance

17:26 when I made the corner the firing as it

17:29 starts to increase

17:31 I can see now I can see the

17:34 sliding glass doors the sliding glass

17:37 doors of blinds or curtains or whatever

17:39 it was was closed

17:40 but I can see because of the darkness

17:42 inside the apartment I can see

17:44 the flashes the muzzle flashes

**Fig. 10:**
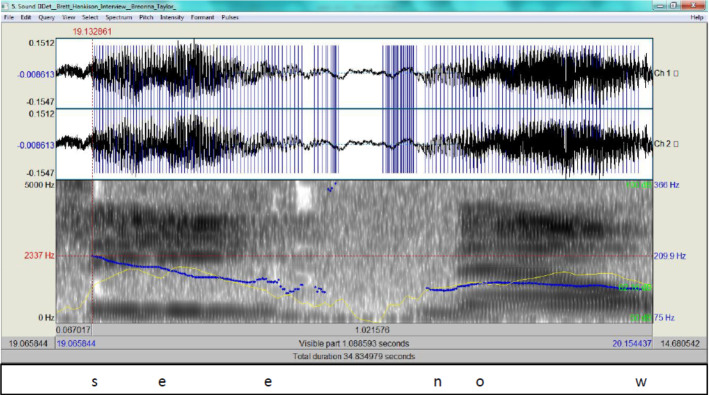
The spectrogram for ‘see now’

Here, Hankison details how the shooting was done by Walker. The repetitions of pronoun ‘I’ reveal his disturbance, how step e in ADCM is being utilized to the full. His spectrogram of the part from 17:31 to 17: 44, where the contradiction between seeing and darkness expresses the heavy cognitive overload, corroborates a fact underlined by Köhnken (2004) and Vrji et al. (2008). The tone is not stable. The spectrogram for ‘I can see now’ is given in Fig. 10.

**Fig. 11:**
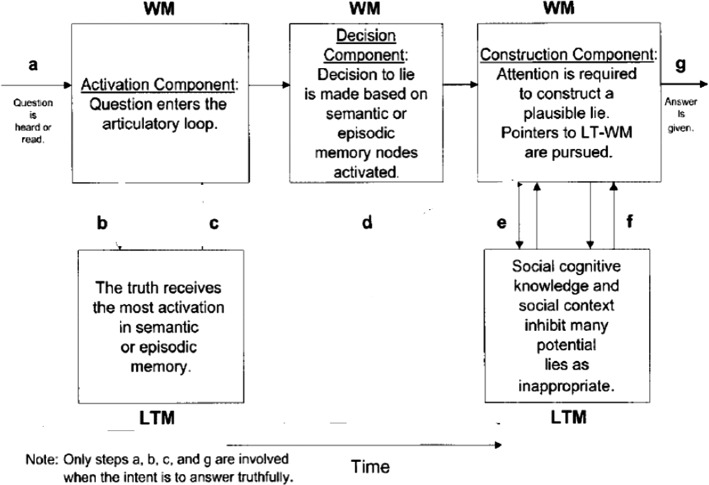
The ADCM for Hankison’s statement in the 2^nd^ episode

It is obvious from Fig. 10 that the account is garbled, as his tone is rising (the pitch contour as an indicator of F0 is 209.9 Hz, which is close to the upperbound 336 Hz) as shown by the blue streaks. An envisaged ADCM of his account can be illustrated by Fig. 11.

According to Fig. 11, the ADCM reveals that Hankison used steps e and f to engage the interrogator in his narrative by means of social cognitive knowledge, where recalling a painful memory may cause agitation. That is why he utilized steps e and f to the fullest, but did not check the veracity of his account by reverting to steps b and c. This renders his account highly dubious.

If matched with Walker’s statement, the dubiety of Hankison’s speech is almost clear:

I’m like scared to death like now we’re seein’ lights and s- stuff. So I was lookin’ around, okay it’s the police and there’s a lot of yellin’ and stuff. So there’s just shooting and like we’re both on the ground and then when all the sh- shots stop I’m, like, panicking. She’s right there on the ground like bleeding and - yellin’.

**Fig. 12:**
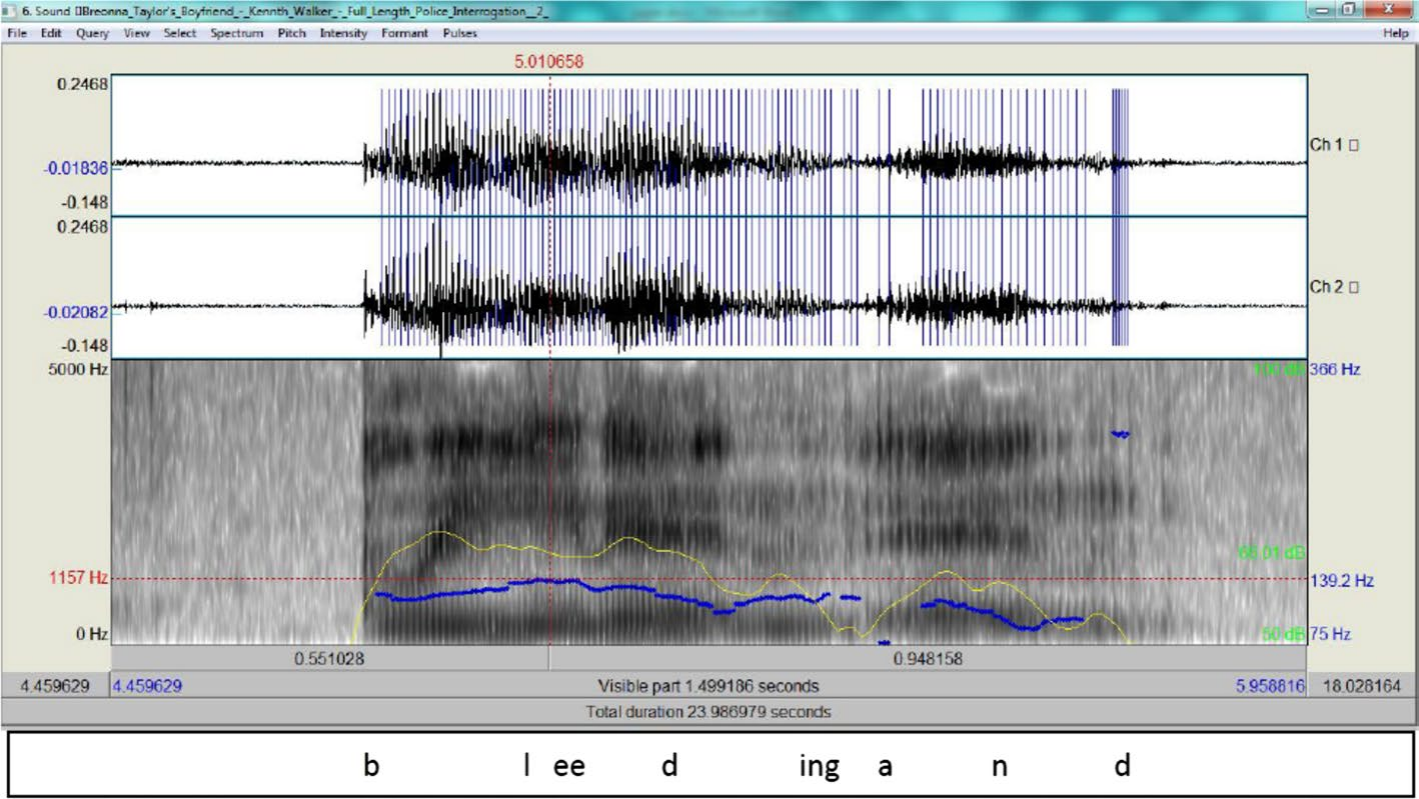
The spectrogram for ‘bleeding and’

Walker states that there was shooting and Hankison also said so, but both did not specify who was firing. This means that one of them is being deceitful, but Walker’s spectrogram shows he is not as disturbed as Hankison, even though the former was describing Breonna’s death. Figure 12 for ‘bleeding and’ shows how his delivery can be deemed more truthful.

In Fig. 12, although Kenneth recalls Breonna’s death, it is clear that the intonation marks no stress in his delivery and the blue streaks (the pitch contour as an indicator of F0 is 139.2 Hz) exhibit how normal the tone is, which runs counter extreme agitation on Hankison’s part when he describes himself being shot at. Again, Hankison’s account is clearly a result of excessive cognitive overload, and the veracity of his description is highly questionable.

### Inconclusively Charged Detectives’ Episodes

This includes the interrogations of both Jonathan Mattingly and Myles Cosgrove, who were reassigned to administrative duties, based on falsifying their accounts. The episodes of their answers, where they spoke about shooting, were examined. Each episode is provided with its timestamps according to the recordings of the interrogation by Sgt. Amanda Seelye.

#### Jonathan Mattingly

Episode 1

12:20 there's a bedroom door on the right

12:22 there's a male and a female

12:25 the male's closest to the door so it's

12:27 to my right

12:28 and as I turn the doorway he's in a

12:32 stretched out position with his hands

12:33 with a gun and as soon as I clear

12:36 he fires boom and uh it was almost like

12:39 at the shooting range where two

12:41 two things flip at the same time you

12:42 gotta shoot no shoot I mean they were

12:44 like shoulder shoulder

**Fig. 13:**
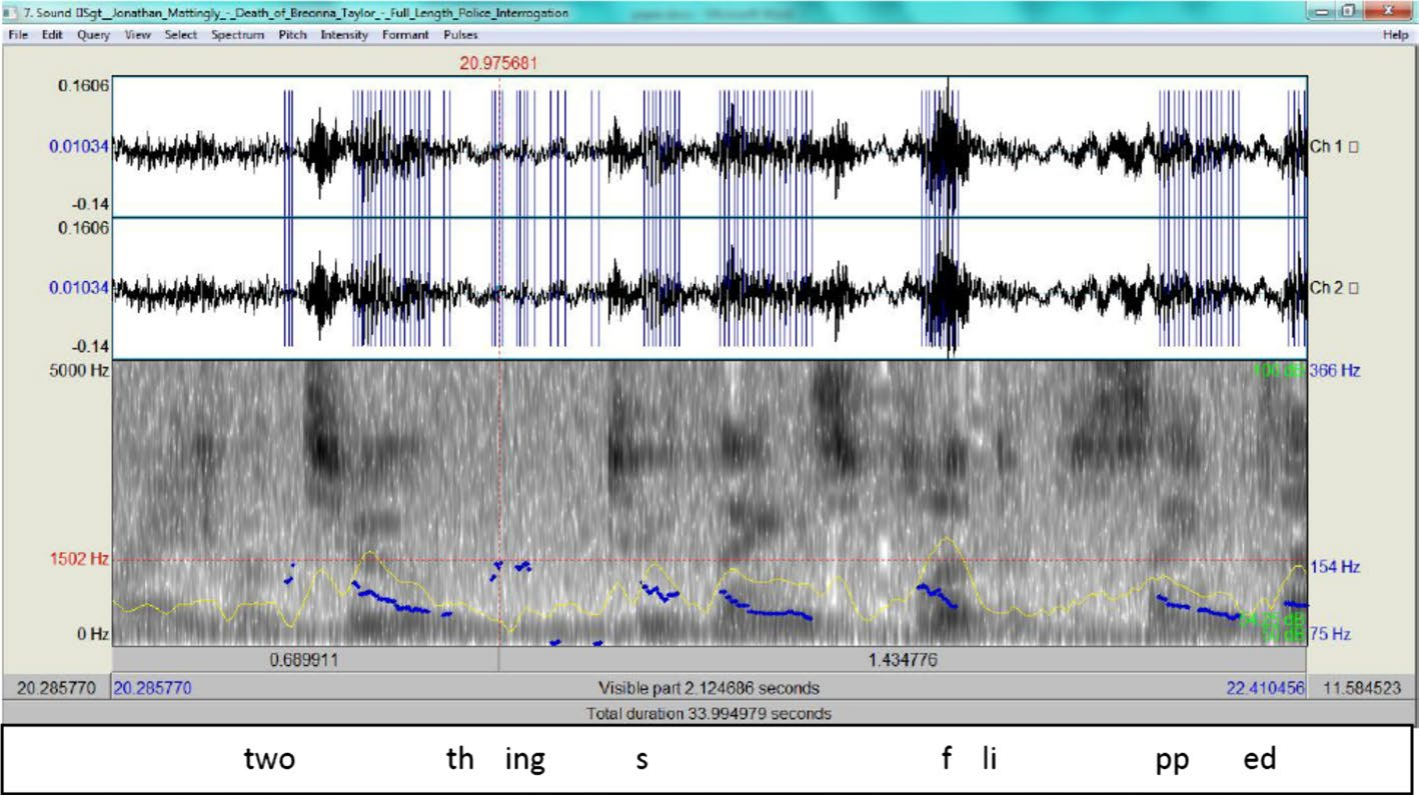
The spectrogram for ‘two things flipped

**Fig. 14:**
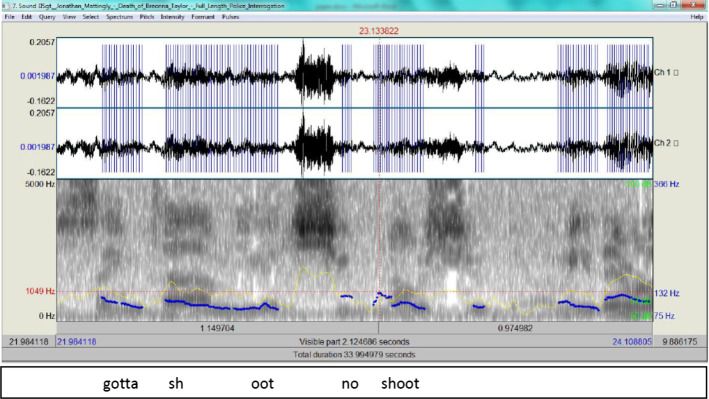
The spectrogram for ‘gotta shoot no shoot’

Two chunks in this episode raise questions as to the veracity of the account. The first is the ‘two things flipped’ while the second is ‘gotta shoot no shoot’. The first does not clarify which is meant by ‘two’: the male and female mentioned before, or the two bullets fired by Kenneth Walker? The second includes a corrective phrase ‘no shoot’, which renders the flow of the account garbled. Although the linguistic aspects of the account point to its deceptive nature, the spectrograms (Figs. 13and 14, respectively) show that the delivery is within normal ranges.

**Fig. 15:**
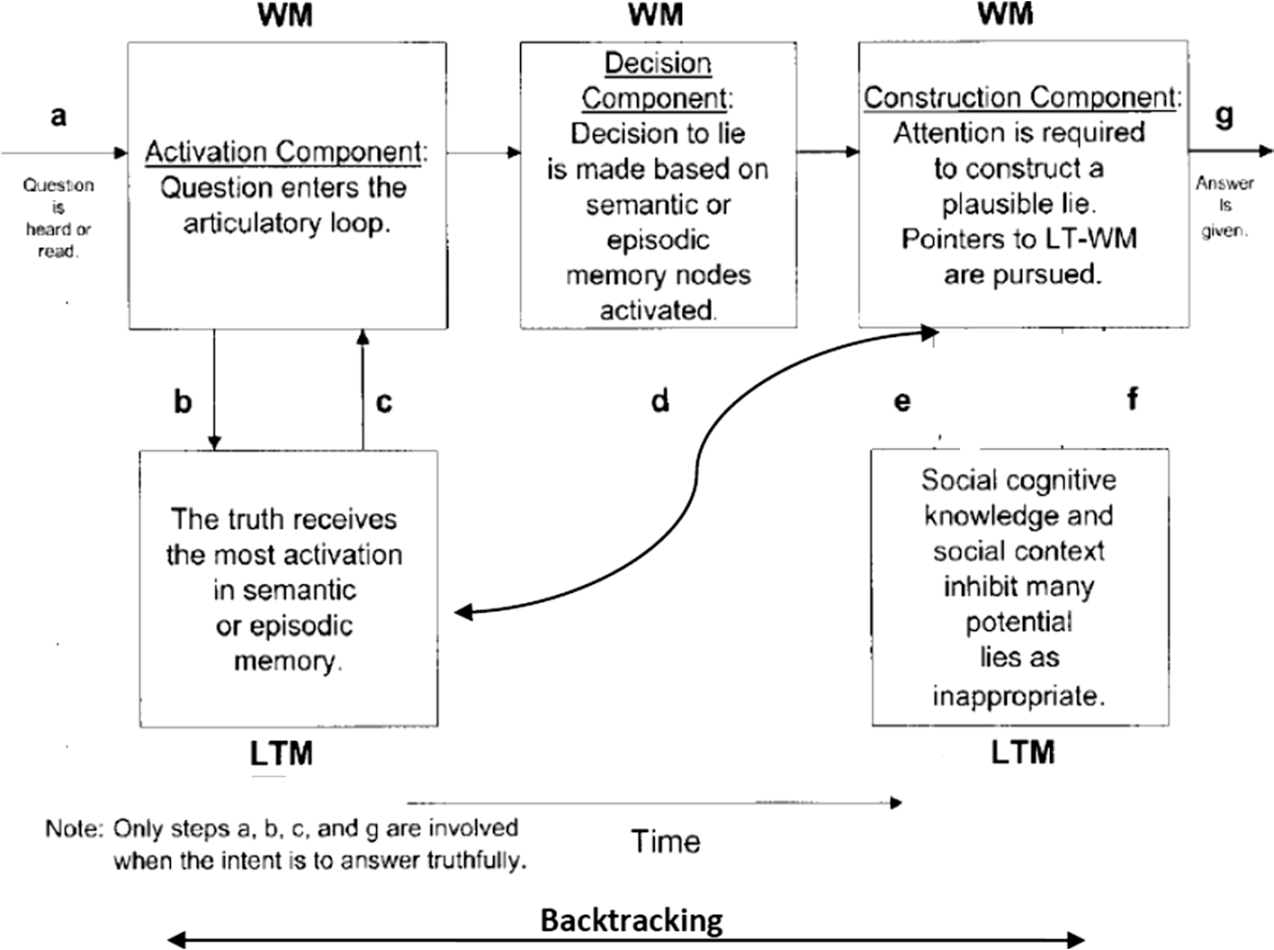
The ADCM for Mattingly’s statement in the 1^st^ episode

What can be gleaned from these two spectrograms (Fig. 13 and Fig. 14) is that when Mattingly recants, his tone rises, but it remains within normal ranges, i.e. 154 Hz and 132 Hz, respectively. His ADCM (Fig. 15) relies heavily on steps b and c and backtracking to provide plausible narratives.

According to Fig. 15, backtracking is clear in his chunk ‘gotta shoot no shoot’, where he revises the whole process to provide the truth.

Episode 2

13:03 and it was like simultaneously boom boom

13:05 boom boom boom

13:06 and then I went back and went down on

13:08 the side of the door and then reached

13:09 around and I think I got two more off

13:12 around the corner of the door and then I

13:14 could really feel the blood in my legs

13:15 so I reached out and felt it my hand was

13:17 full blood and I knew it

13:18 hit my femoral

**Fig. 16:**
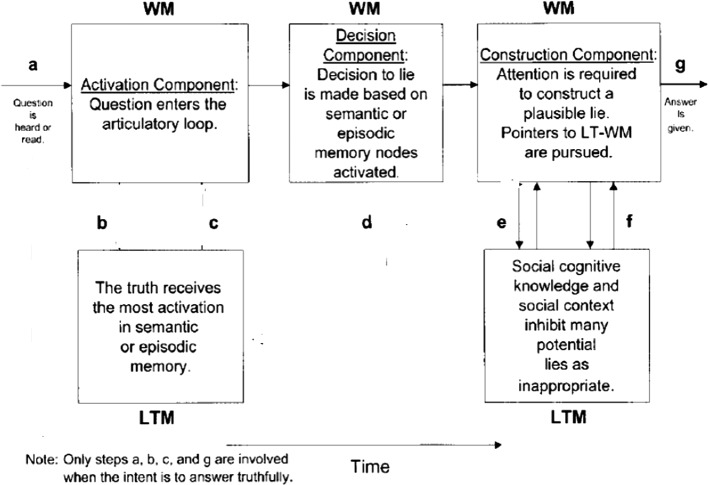
The ADCM for Mattingly’s statement in the 2^nd^ episode

Here, Mattingly spoke about the encounter of shooting, but he did not specify who was firing. Yet it is no sense to presume that his fellow detectives were firing at him. He meant Kenneth Walker, though the latter stated that he fired only once. Mattingly was inserting a lie among a number of truth accounts, and this points to his ADCM operating on steps a, d, e, f, and g (Fig. 16).

**Fig. 17:**
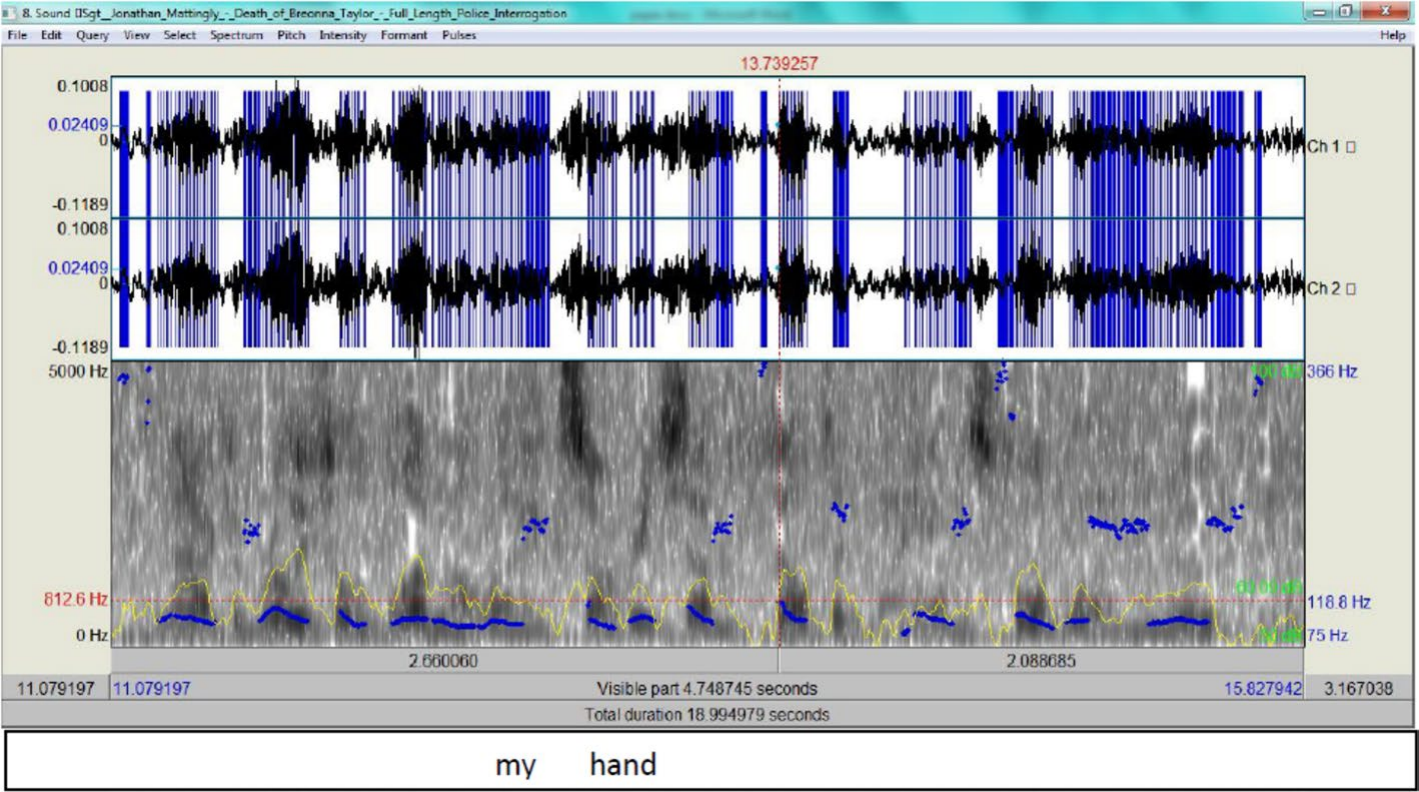
The spectrogram for ‘my hand’

His spectrogram (Fig. 17) exhibits normal F0, but the tone slightly rises (i.e. 118.8 Hz) as he speaks of his hand stained with blood.

Episode 3

15:00 and I got to get that way but I do

15:03 remember when I trip

15:04 by the time I I went out and I stepped

15:06 off the curb and tripped over him

15:08 I remember as soon as I hit the ground

15:09 holster I could hear all sudden boom

15:12 boom boom boom boom boom

15:13 several shots at that point it seemed

15:14 like that

**Fig. 18:**
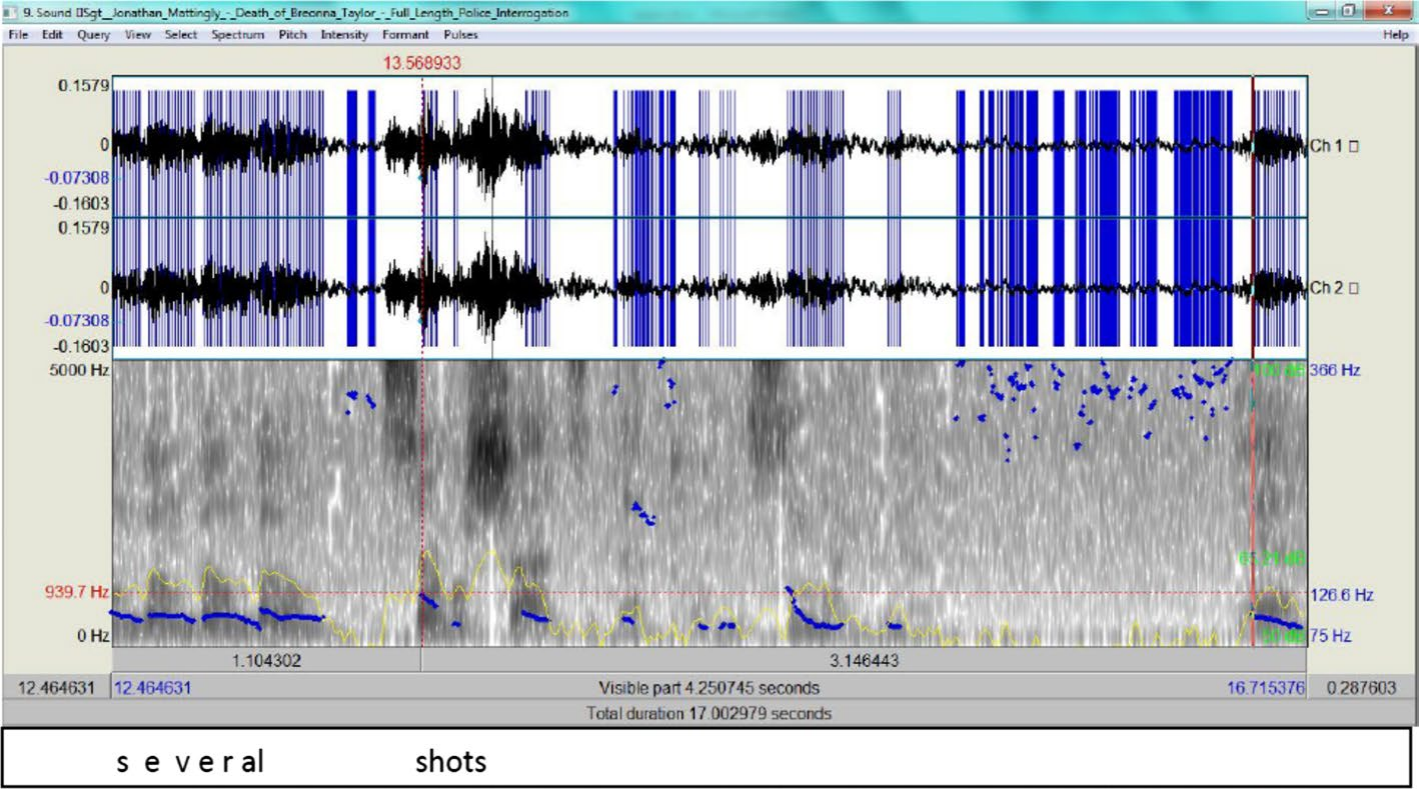
The spectrogram for ‘several shots’

Mattingly was again asserting the assumption that many bullets were being fired, but did not specify who was shooting. Maybe it was Hankison wantonly shooting. His unclear account is also within normal F0 ranges as he mentions a number of booms, then guards off by stating that they were ‘several shots’ (Fig. 18).

**Fig. 19:**
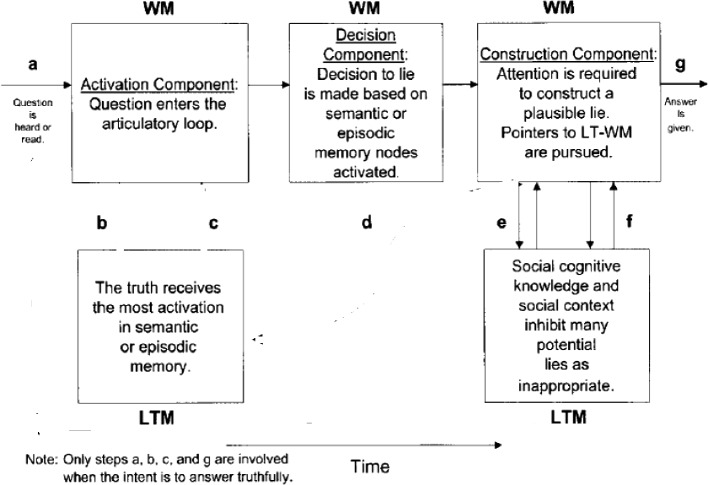
The ADCM for Mattingly’s statement in the 3^rd^ episode

In Fig. 18, the pitch contour rises slightly to 126.6 Hz when clarifying they were several shots. Mattingly is clearly making best use of steps e and f in ADCM (Fig. 19).

His reliance on the cognitive context is clear in his insertion of ‘several shots’ after the series of ‘booms’ he uttered. He was trying to divert the interrogator’s attention from counting the number of booms by guarding off.

Episode 4

Q: 34:26 okay and then you had mentioned you said

34:28 that the guy was stretched

34:30 out what did you mean by that

A: 34:32 both hands pushed out

34:33 in a in fighting shooting stance

**Fig. 20:**
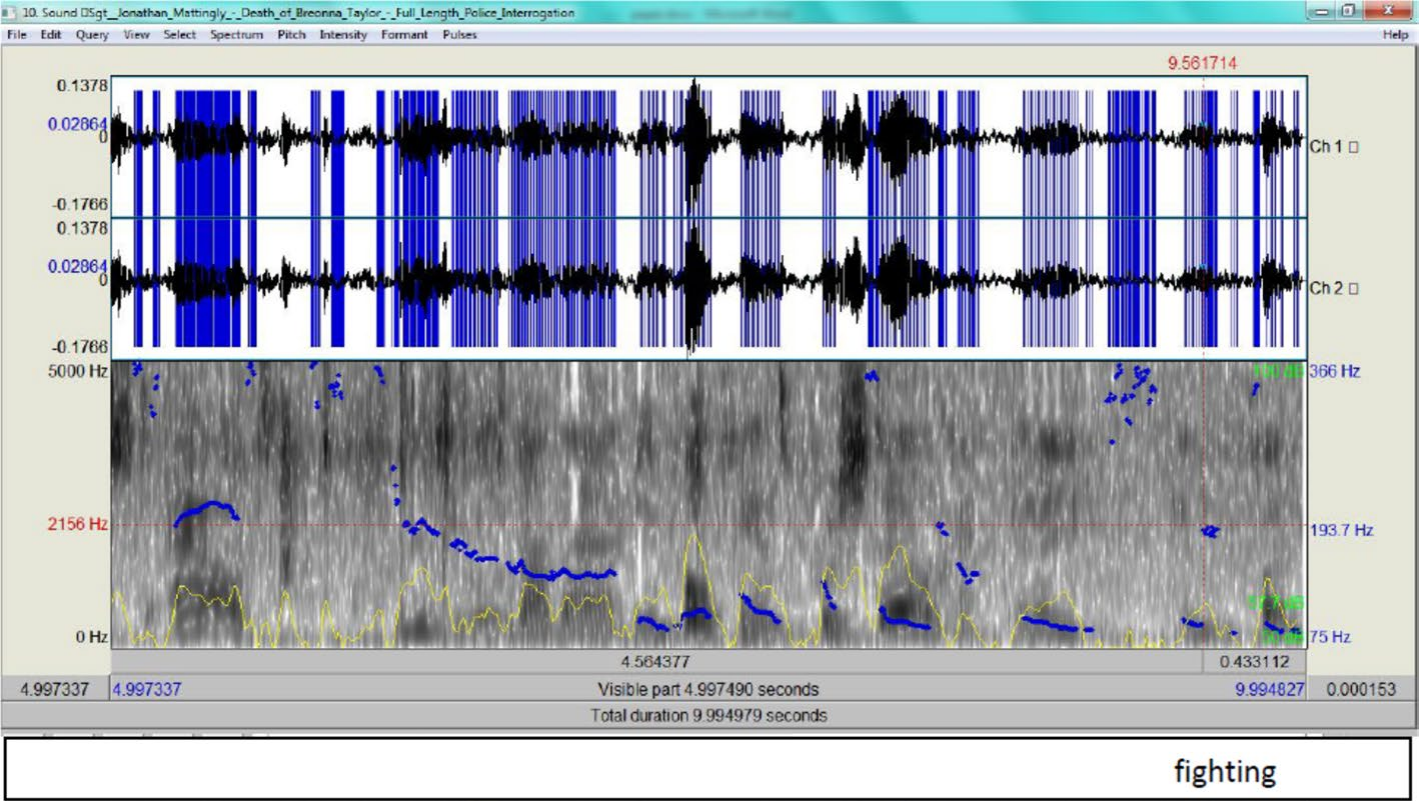
The spectrogram for ‘fighting’

In this exchange between the interrogator and Mattingly, the RT of Mattingly reveals that he was in control and not lying, i.e. 311 ms. Walczyk et al. (2003) theorized that when the truth is told, a response would be produced in less than 400 ms. However, the same attitude of giving two different descriptions for the same action is maintained by Mattingly when he states that ‘in a in fighting shooting stance’. His spectrogram shows a sudden rise in intonation when uttering ‘fighting’ (Fig. 20).

According to Fig. 20, the F0 signals 193.7 Hz which is again completely normal. This renders Mattingly’s account unclear, since linguistically, he is disfluent, but acoustically his delivery is normal. This tallies with Schwandt (2006) who found that a longer response delay and more speech errors indicate that deceptive senders are relying less on automatic recall of a memory, and are focused more on thinking and self-monitoring.

#### Myles Cosgrove

After police used a rod to ply open Breonna Taylor's front door, Taylor's boyfriend, Kenneth Walker, fired one shot from his legally licensed gun, wounding Sgt. Jonathan Mattingly. In return, Cosgrove fired 16 times.

A number of episodes from Cosgrove’s account are analyzed to see whether he was delivering deceptive speech or not.

Episode 1

18:02 I believe at one point I'm standing on

18:04 this person

18:05 that is below me I know that someone has

18:09 been shot that john has been injured

18:14 I continue to see these this blinding

18:17 light

18:17 these vivid white flashes and I

18:21 see this darkness in front of me

18:24 followed by

18:29 and this is hard for me to explain

**Fig. 21:**
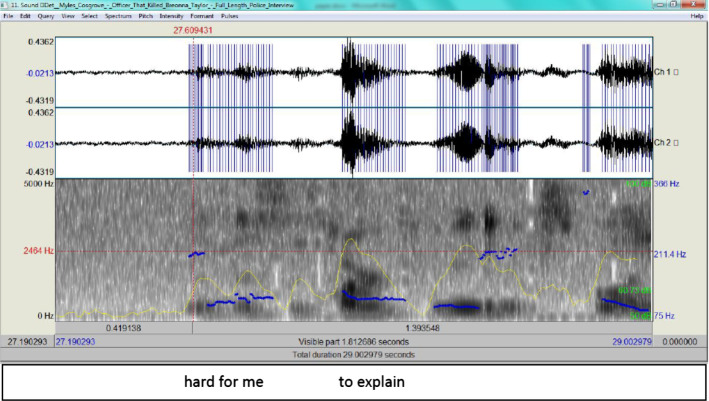
The spectrogram for ‘hard for me to explain’

Cosgrove employed the same technique of stating nothing definite adopted by Mattingly. Here, he stated that he was crushing someone without asking who: probably it was Mattingly, which he later asserted (cf. from 53:02 to 53:08 min). He then went to speak about the seeing light. The problem with his discourse is that he was indifferent to the person whom he was treading on and started to concentrate on the light. Feeling illogical, he said it was difficult for him to explain. His pause before saying ‘this is hard for me to explain’ is almost 3.750 s. This is more than triple the time to fabricate a lie. Moreover, his F0 is high according to the spectrogram in Fig. 21.

In Fig. 21, the pitch contour rises to 211.4 Hz when starting to utter the word ‘hard’. Cosgrove is highly agitated, which renders his veracity at stakes.

Episode 2:

Q:54:16 I mean I don't want to say cans-answer for you but in

54:17 just a very short period of time and

54:19 you guys are almost firing at the same

54:21 time or is it

A:54:23 I I don't know I just see this

54:27 this flash this goody flash and this

54:31 distorted shadowy figure

**Fig. 22:**
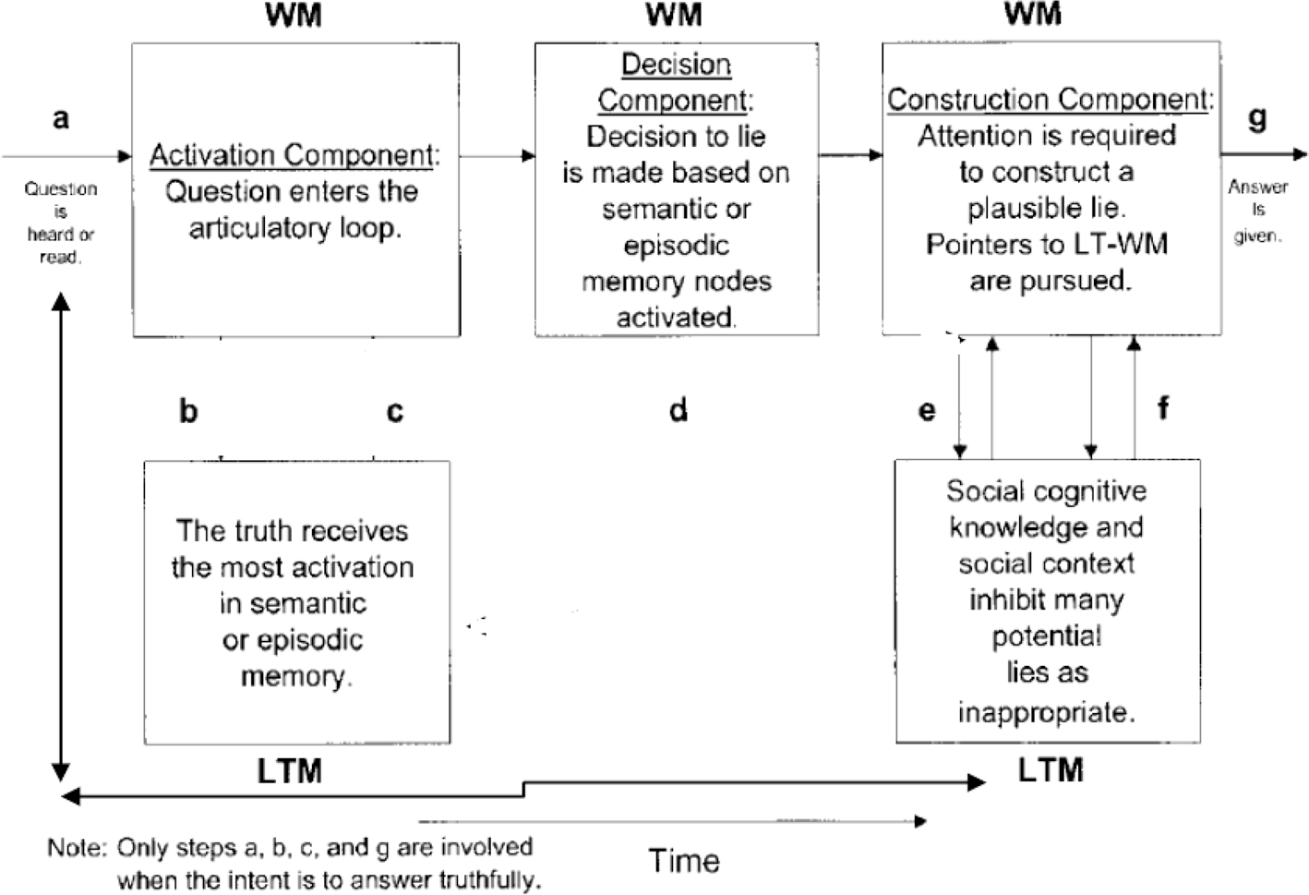
The ADCM for Cosgrove’s statement in the 2^nd^ episode

In this exchange, a male interrogator asked Cosgrove whether someone else than the detectives was firing at a very short span of time, possibly Kenneth Walker, but Cosgrove took considerable time to answer. Most probably, he fabricated a lie, since it took him an RT of 646 ms to answer, which is commensurate with ADMC findings. Yet his F0 remains normal at this juncture (Fig. 22).

In Fig. 22, Cosgrove consumed a lot of time, since he repeated the whole process twice, but did not attempt steps b and c. Moreover, his insertion of ‘I don’t know’ casts doubt on his veracity (Bachenko et al., 2008). That is why he produced a dubious account.

Episode 3:

Q: 54:48 but you knew that john had been shot and you– you knew that John had discharged his weapon

A: Yes I knew that clearly that John had been shot

**Fig. 23:**
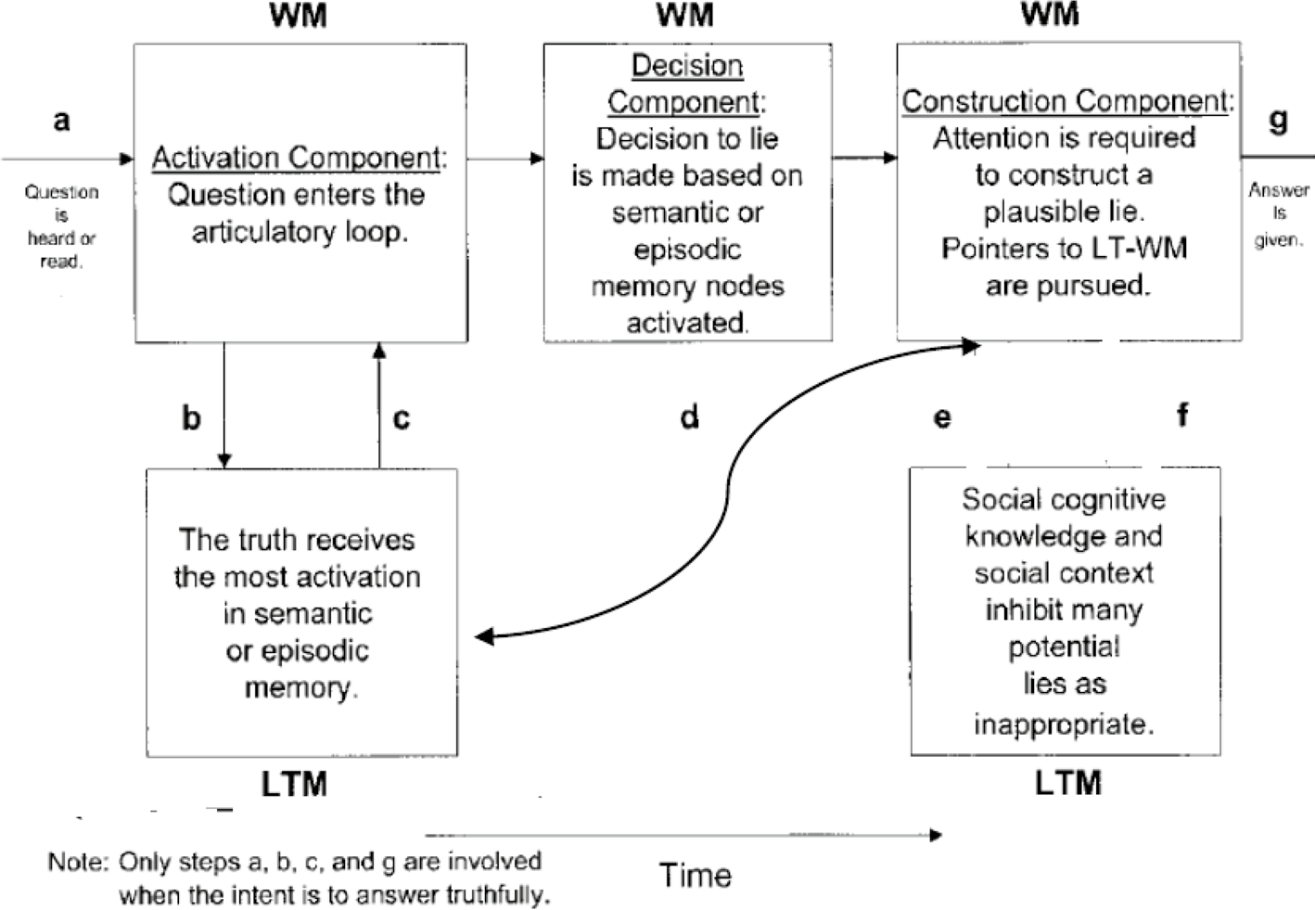
The ADCM for Cosgrove’s statement in the 3^rd^ episode

In this exchange, Cosgrove did not wait until the male interrogator finished his question but answered in the middle of the conversation. In fact, he did not answer the question but revealed what he intended to say. The question was whether John Mattingly *had shot*, but Cosgrove’s answer was he had been sure that John Mattingly *had been shot*. Linguistically, Cosgrove provided the wrong answer, but acoustically, his RT is within normal limits, i.e. 113 ms. What he did can be envisaged according to ADMC in Fig. 23.

The ADCM in Fig. 23 reveals that he was certain about his answer, so he did take time to rethink the question. He looped from steps b and c to g directly. Yet he focused on his semantic memory and answered wrongly but truthfully.

### An Overall Analysis of RT

This overall analysis aims to integrate the numerical/statistical dimension into the present methods adopted. The RT is a major indicator in lie detection, and occupies a special overarching place in ADCM. Table 4 summarizes the mean RT of each of the detectives in addition to Kenneth Walker.

These results reveal that Hankison can be easily identified as not telling the truth, while the rest were very close to dubiety except Kenneth Walker. This finding is tallied with Walczyk et al’s. (2003) in addition to Bird (2018). A more comprehensive picture can be achieved if the RT means are compared with the durations of each detective’s accounts:

The table provides the detectives’ speaking times only. The correlation between the RTs and the lengths is calculated using Pearson Correlation Coefficient:

The results of the Pearson correlation indicates that there is a non-significant very small negative relationship between X and Y, (*r*(2) = 0.335, *p* = 0.665). The p-value equals 0.665, ( P(x ≤ -0.5028) = 0.3325). It means that the chance of type I error is too high: 0.665 (66.5%). Yet The test statistic **T** equals -0.5028, which is in the 95% region of acceptance: [-4.3027: 4.3027].

The results explain that a very small negative correlation is found which sheds light on the high reliability of RT, yet this scant non-significant can be attributed to the inconclusive charges against Mattingly and Cosgrove. The veracity of their accounts is still dubious, and no clear conclusion can be reached.

**Table 4:** Pearson correlation coefficient for the RTs and the detectives’ account lengths

Parameter	Value
Pearson correlation coefficient (r)	**−0.335**
P-value	0.665
Covariance	−399,616.6667
Sample size (n)	4
T-value	−0.5028

## Conclusions

It can be concluded that the deception detected according to the approach proposed here is tallied with the actual punitive measures taken against the detectives in the event of shooting Breonna Taylor. The episodes chosen and how they are analyzed exhibit that the modified ADCM along with the acoustic dimension provide a clear picture of cognitive load management in the course of constructing and producing lies.

In the case of Brett Hankison, there is a persistence to fabricate certain lies in order to show that Kenneth Walker, Breonna’s boyfriend, let off several shots which urged the detective(s) to shoot in return. However, the fact is that Walker released only one shot from his legally licensed handgun as a warning. This did not require the detectives, especially Hankison, to shoot wantonly and injure Breonna to death. Hankison’s outputs in the episodes examined were mostly agitated and his ADCMs omit the truth checking phase, i.e. a and b steps. This agitation is exemplified by the spectrograms which show how his F0 is within normal ranges at times but other times the pitch levels rise unjustifiably, causing his statements to be highly questionable.

As for Jonathan Mattingly and Myles Cosgrove, their statements are mostly unclear in terms of ADCM and F0. However, their befuddlement and disfluencies render their confessions vague at times. These confessions reveal that they agree with Hankison hearing several shots, but they did not specify who was firing: they left the answer open to guess that Walker was the one firing while, in fact, both Hankison and Mattingly fired several times. Perhaps Cosgrove was the most stable of them, keeping a steady F0 and a reasoned ADCM most of the time.

An examination of the RTs in the episodes investigated also reveals that Hankison’s statements exceeded that normal range of producing truthful accounts. His RTs tally with what is reported in the literature, particularly in view of ADCM, with regard to the long duration consumed to produce lies. Mattingly and Cosgrove, on the other hand, maintained RTs very close to deceptive outputs, but they did not exceed normal limits.

The above conclusions point to the fact that the charges and punitive actions taken against the accused detectives in question are consistent with the findings obtained here, particularly in the case of Hankiosn. Yet Mattingly’s and Cosgrove’s accounts appear to be closer to deception than truth-telling, which made the exact determination of their lying hard to state as a fact.

It is noteworthy that the present study is confined to the investigation of the episodes where shooting is concerned and to the parties involved in the shooting, and most of them are either sacked or give administrative duties. Other studies can handle more eyewitnesses’ accounts or engage in specialized statistical analysis to determine the interplay of figures and acoustics in the process of differentiating truthful from deceitful discourse.

## Footnotes

1 Bird (2018) maintains that longer reaction time (RT) is associated with a proportionate increase in cognitive load, and is growing evidence that RT is a reliable measure of the cognitive load.

2 In fact, the model was modified in 2014 by Walczyk et al. The new component added was Action, so the model’s name was changed to Activation-Decision-Construction-Action. Yet the present paper does not adopt the modification, since it focuses on cases where the speaker leaves a certain impression on the receiver and prefers to withhold a lie, which is not part of the data analyzed here. The focus here is on how the model as a whole can be modified to allow for cognitive flexibility.

3 Kintsch's model is predominantly is built on two focal processes, i.e. construction and integration. The constructive phase occurs according to a textbase which is made up of propositions or concepts. These propositions are like nodes in a network and are connected to each other. They have a 'strength value' (Kintsch's term) ranging from zero to negative. As Kintsch claims, there are two ways to look at propositions or nodes: either as ' a portion of a general knowledge network' or as a base for a discourse (p.165). Integration, on the other hand, is a sequence to construction, where node activation 'spreads around until the system stabilizes' (p.168). However, according to Kintsch (p.170), the model is not interactive, and priming is not involved. Meaning is thus constructed for the word in context.
